# Discriminating Diseases Mimicking Normal-Tension Glaucoma (NTG) from NTG

**DOI:** 10.3390/jcm13216585

**Published:** 2024-11-01

**Authors:** Hee-Kyung Ryu, Seong-Ah Kim, Hee-Jong Shin, Chan-Kee Park, Hae-Young Lopilly Park

**Affiliations:** Department of Ophthalmology, Seoul St. Mary’s Hospital, College of Medicine, The Catholic University of Korea, Seoul 06591, Republic of Korea; heekyung130@naver.com (H.-K.R.); sah900216@naver.com (S.-A.K.); shinhj01@naver.com (H.-J.S.); ckpark@catholic.ac.kr (C.-K.P.)

**Keywords:** NTG, neuro-ophthalmologic disease

## Abstract

**Background/Objectives:** The aim of this study was to identify the most reliable ocular exam and establish a threshold for deciding whether to perform neuroimaging in order to screen for diverse diseases other than normal-tension glaucoma (NTG). A retrospective, observational, comparative study was used. **Methods:** In total, 106 individuals with atypical features of NTG who underwent glaucoma assessments and contrast-enhanced MRI of the brain or orbit were included. The criteria for atypical NTG included the following: (1) unilateral normal-tension glaucoma, (2) visual field (VF) damage inconsistent with optic disc appearance, (3) fast VF progression, (4) worsening of visual acuity, (5) optic disc pallor, (6) scotoma restricted by a vertical line, and (7) central scotoma. Glaucoma evaluations included measurements of visual acuity, intraocular pressure, central corneal thickness, axial length, cup–disc ratio, retinal nerve fiber layer (RNFL) thickness, ganglion cell–inner plexiform layer (GCIPL) thickness, mean deviation (MD), and visual field index (VFI). Statistical analyses involved independent *t*-tests, receiver operating characteristic (ROC) curves, and area under the curve (AUC) in order to differentiate neuro-ophthalmological conditions from NTG, compare the diagnostic power of each factor, and determine the cut-off value. **Results:** Relatively fewer diagnoses of non-glaucomatous diseases were associated with unilateral NTG, the worsening of VA, and central scotoma. Factors such as rapid visual field progression, optic disc pallor, and scotoma restricted by a vertical line had a relatively higher diagnostic rate of non-glaucomatous diseases. There were significant differences in average RNFL and GCIPL thicknesses at the nasal quadrant between NTG and NTG-mimicking conditions. Only the GCIPL thickness at the nasal quadrant had reliable power for discriminating between neuro-ophthalmological disease and NTG. For the GCIPL thickness at the nasal quadrant, the AUC was 0.659, and the cut-off value was 65.75. **Conclusions:** When deciding whether to proceed with imaging, such as carrying out an MRI test, for NTG patients with atypical NTG characteristics, it would be advisable to consider the nasal sector cut-off value of GCIPL thickness.

## 1. Introduction

Normal-tension glaucoma (NTG) comprises open-angle glaucoma with intraocular pressure (IOP) within the statistically normal range (≤21 mmHg), in addition to specific features observed in the glaucomatous optic disc and visual field and without other known ocular or systemic disorders [1,2,3]. The distinction between NTG and other systemic conditions is crucial, in order to exclude life-threatening conditions [3,4,5]. However, routine neuroimaging for patients with presumed NTG is a debated topic because of its low ratio of intracranial pathology detection and low cost-effectiveness [6,7].

In addition, myopia has recently been increasing, and the number of NTG cases with atypical test results is also increasing, which renders the diagnosis of NTG more challenging in countries with a high prevalence of myopia [8,9,10,11]. Therefore, in such situations, having additional reference metrics from glaucoma exams beyond the known characteristics of atypical NTG would be helpful in deciding whether to perform neuroimaging.

In previous studies exploring the relationship between glaucoma exam results and neuro-ophthalmologic diseases, it has been noted that the thinning of the ganglion cell–inner plexiform layer (GCIPL) may be detected before the loss of the retinal nerve fiber layer (RNFL) in cases of chiasmal compression, with nasal GCIPL being associated with this specific condition [12,13]. Another study targeting various neuro-ophthalmologic diseases reported that, aside from age, no significant differences were observed between NTG and neuro-ophthalmologic diseases in glaucoma exam results. The glaucoma exam results included the cup–disc ratio, RNFL thickness, and mean deviation of the visual field exam, but did not further subdivide RNFL and GCIPL into superior/inferior/nasal/temporal quadrants [14].

While various atypical NTG findings that may warrant consideration of imaging exams are known to locate diverse NTG-mimicking neuro-ophthalmological diseases, there has been no study that has analyzed glaucoma exam metrics in detail [3,12,14,15]. The purpose of this study is to find the most reliable glaucoma exam and the standard value that can serve as a reference in deciding whether to perform neuroimaging to screen for diverse diseases other than NTG. Although its diagnostic power is lower than that of neuroimaging tests such as MRI, it is meaningful in providing guidance on whether to proceed with neuroimaging for atypical NTG patients in a real clinical setting.

## 2. Method

### 2.1. Subjects

This study was a retrospective one-center study carried out in 2015–2022 at the Department of Ophthalmology, Seoul St. Mary’s Hospital. This was approved by the Institutional Review Board of Seoul St. Mary’s Hospital (KC24RASI0368). All relevant tenets of the Declaration of Helsinki were followed.

The inclusion criteria for this study comprised patients who, among those visiting the clinic either for a diagnosis of NTG received at another hospital or for regular NTG check-ups, underwent brain evaluation due to atypical features of NTG. Each participant underwent a comprehensive glaucoma assessment followed by either contrast-enhanced brain MRI or orbit MRI.

NTG was labeled when typical glaucomatous disc change (cup–disc ratio of 0.6 or greater), RNFL defects at the superotemporal or inferotemporal regions with corresponding visual field defects, and open anterior chamber angles were associated with an IOP value that was constantly below 21 mmHg. Atypical NTG features that resulted in neuroimaging were the following: (1) unilateral normal-tension glaucoma (the fellow eye shows no signs of glaucoma in all aspects of glaucoma evaluation); (2) VF damage that is inconsistent with optic disc appearance; (3) fast visual field progression (1.5 dB per year or more); (4) worsening of VA (≤0.5) (worsening of the visual acuity of at least 2 lines in the Snellen chart connected neither with lens nor retinal pathology); (5) optic disc pallor; (6) scotoma restricted by a vertical line; and (7) central scotoma.

Glaucoma examinations included the following: the measurement of best-corrected visual acuity; the measurement of intraocular pressure via noncontact tonometry; the measurement of the central corneal thickness; axial length measurement; slit-lamp microscopy; color disc and red-free retinal nerve fiber layer (RNFL) photography (Canon, Tokyo, Japan); Humphrey VF examination (24–2 Swedish Interactive Threshold Algorithm Standard program; Carl Zeiss Meditec, Dublin, CA, USA); and Cirrus optical coherence tomography (OCT) (Carl Zeiss Meditec, Dublin, CA, USA).

Patients with detectable eye pathology that would imply a diagnosis of secondary glaucoma (such as neovascularization, pseudoexfoliation, or pigment dispersion syndrome) were excluded from the NTG diagnosis from the outset [16,17,18]. Several eye pathologies that can mimic NTG with normal intraocular pressure were also excluded. For example, conditions such as coloboma of the optic nerve head (OHN), pits, and ONH oblique insertion, as well as autosomal dominant optic atrophy, were ruled out by examining the optic disc shape in disc photos during the NTG diagnosis process. Additionally, a history of steroid use, previous trauma or surgery that could have caused prior elevated IOP, and hemodynamic crisis were excluded via patient history verification. Anterior optic ischemic neuropathy (AION) was also excluded during the initial patient history intake. Patients meeting any of the following criteria were also excluded: a cataract with a LOCS III grade higher than grade 3; a history of any retinal disease, including myopic retinopathy or other retinal complications; and if only one of both eyes had atypical NTG features, only that eye was selected and the other eye was excluded.

### 2.2. Ophthalmological Examination

The Cirrus HD-OCT (software version 11) was utilized for capturing scans of the macula using the Macular Cube 512 × 128 protocol and optic disc with the Optic Disc Cube 200 × 200 protocol to obtain mGCIPL and cpRNFL measurements. The image quality was assessed based on the signal strength index, and it ranged from 0 to 10 arbitrary units, as automatically determined via the OCT instrument. Only scans with signal strength values above 6 units were included in the analysis. The recognition algorithm was used to calculate the RNFL thickness from the vitreoretinal interface to the IPL (inner plexiform layer) by drawing a 1.73 mm diameter circle centered on the optic nerve’s head in the optic nerve cube scan. The RNFL thickness was measured both on average and at 4 quadrants around the optic nerve, including the temporal, superior, nasal, and inferior regions. Another algorithm measured GCIPL thicknesses and the distance between the RNFL and IPL outer boundaries around the fovea. The average and sectoral (superotemporal, superior, superonasal, inferonasal, inferior, and inferotemporal) mGCIPL thicknesses were analyzed.

Visual field exams were considered reliable if fixation losses were less than 20% and false-positive and false-negative rates were less than 15%. The MD (mean deviation) represents the average deviation in the total visual field’s sensitivity from age-adjusted normal values, and these are expressed in decibels (dB), indicating the overall severity of visual field impairment. The VFI (visual field index) is a percentage score that reflects the overall visual field loss. It is calculated by comparing the patient’s visual field to a normative database, with 100% representing a completely normal visual field.

The parameters, such as MD/RNFL thickness, MD/GCIPL thickness, VFI/RNFL thickness, and VFI/GCIPL thickness, relate the overall visual field impairment to the structural integrity of the optic nerve. In neuro-ophthalmological diseases, unlike in NTG where optic nerve damage and corresponding visual field impairment typically occur, a discrepancy can exist [19,20]. Over time, due to retrograde trans-synaptic degeneration, the damage to the optic nerve and visual field becomes aligned [21]. However, initial mismatches can be a key characteristic.

### 2.3. Neuro-Imaging Acquisition

Only patients who underwent a contrast-enhanced brain or orbit MRI and received radiological interpretation were selected. All MRI tests were performed on a 3 T scanner (Magnetom Vida, Siemens Healthcare, Erlangen, Germany) and a 64-channel head coil. Brain and orbit MRI was carried out in 3 planes (axial, coronal, and sagittal) before and after the intravenous administration of a contrast agent. Both MRI scans employed the fast spin echo technique to generate T1- and T2-weighted images.

Orbit MRI was obtained alongside MRI of the brain. The protocol included T1- and T2-weighted images with a slice thickness of 2.5 or 3.0 mm, covering the entire brain. Additionally, thin-cut axial T1-weighted and axial and sagittal T2-weighted images with a 0.8 or 1 mm slice thickness were acquired for detailed brain imaging.

The brain MRI included T1- and T2-weighted images with a slice thickness of 5 mm, axial susceptibility-weighted images with a 2 mm slice thickness, and T1-weighted images with a 1 mm slice thickness.

### 2.4. Statistical Analyses

An independent *t*-test was used to compare differences between NTG and neuro-ophthalmological conditions. Receiver operating characteristic (ROC) curves were constructed to assess the ability of each factor to discriminate patients with neuro-ophthalmological disease other than NTG and determine the optimal cut-off value. Area under the curves (AUCs) were also used to represent the diagnostic ability. *p*-values of <0.05 were considered statistically significant. Statistical analyses were performed using the SPSS version 24.0 software (SPSS, Chicago, IL, USA), MedCalc version 22.014 (MedCalc Software, Ostend, Belgium), and statistical software package R version 4.3.1 (The R Project for Statistical Computing, Vienna, Austria).

## 3. Results

### 3.1. Patient Characteristics

The NTG patients included consisted of 106 persons: 71 (67.0%) female and 45 (42.5%) male individuals. The mean age of patients was 52 years old (ranging from 17 to 81 years). The demographic and clinical characteristics are summarized in Table 1 and Table 2. After neuroimaging, the results of 27 (25.5%) patients were diagnosed as having neuroophthalmological diseases other than NTG. Among the pathological results other than NTG, the most frequent brain diseases were intracranial meningiomas and old infarctions observed in five patients (18.5%). Diagnosed brain diseases using neuroimaging were mostly diseases causing compressive optic neuropathy (*n* = 12, 44.44%), including meningioma (*n* = 5), pituitary macroadenoma (*n* = 2), cavernous hemangioma (*n* = 1), aneurysm (*n* = 1), Rathke cleft cyst (*n* = 1), pineal cyst (*n* = 1), and empty sella syndrome (*n* = 1) (Table 1).

Table 2 compares ocular characteristics between NTG and NTG-mimicking neuro-ophthalmological conditions. There were significant differences in average RNFL and GCIPL thicknesses at the nasal quadrant. (*p* < 0.05 and *p* < 0.05, respectively).

Table 3 outlines the reasons why patients underwent neuroimaging and the percentage of significant results other than NTG. The total sum exceeds 100% as there were some patients recommended for neuroimaging for more than two reasons. The most common reason was unilateral NTG, which was reported in 64 cases, comprising 59.81% of the total. In this category, 18 patients, or 28.13% of those who underwent imaging for this reason, were found to have neuro-ophthalmological diseases. Unilateral NTG exhibited a relatively lower diagnostic rate compared to other factors. Visual field (VF) damage that was inconsistent with optic disc appearance was the second most common reason, and this was noted in 42 patients (39.25% of the total), with neuro-ophthalmological conditions identified in 33.33% of these cases. Other reasons included rapid visual field progression (4.67%), with 40% exhibiting neuro-ophthalmological conditions; worsening of visual acuity (34.58%), with 29.73% having the associated conditions; optic disc pallor (34.58%), with 35.14% having related conditions; scotoma restricted by a vertical line (24.30%), with 38.46% having neuro-ophthalmological conditions; and central scotoma (17.76%), with 21.05% having related conditions. The proportion of diagnosed neuro-ophthalmological diseases varied depending on each factor.

### 3.2. Determination of the Optimal Cut-Off Value for Discriminating Neuro-Ophthalmological Conditions from NTGs

**Figure 1:**
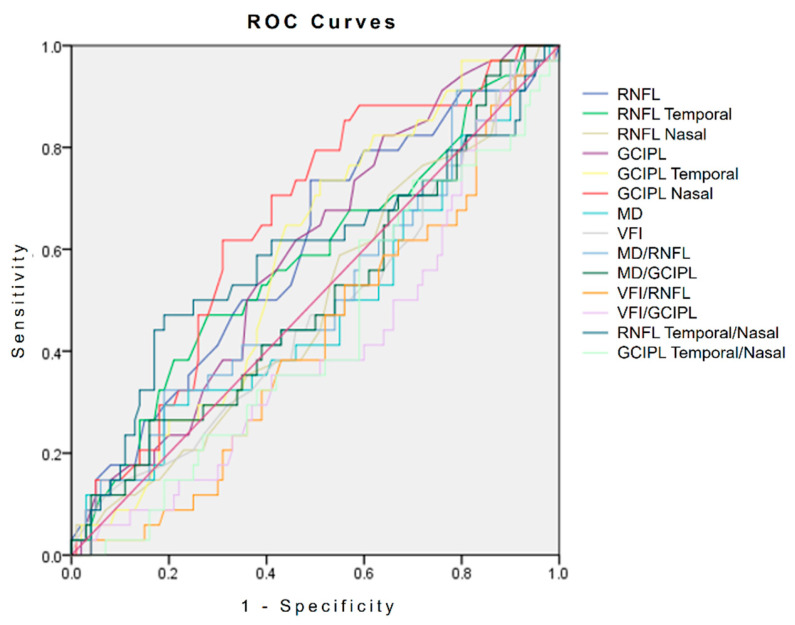
Receiver operating characteristic (ROC) curves showing the performance of the best individual parameters in discriminating neuro-ophthalmological conditions and NTG.

**Figure 2:**
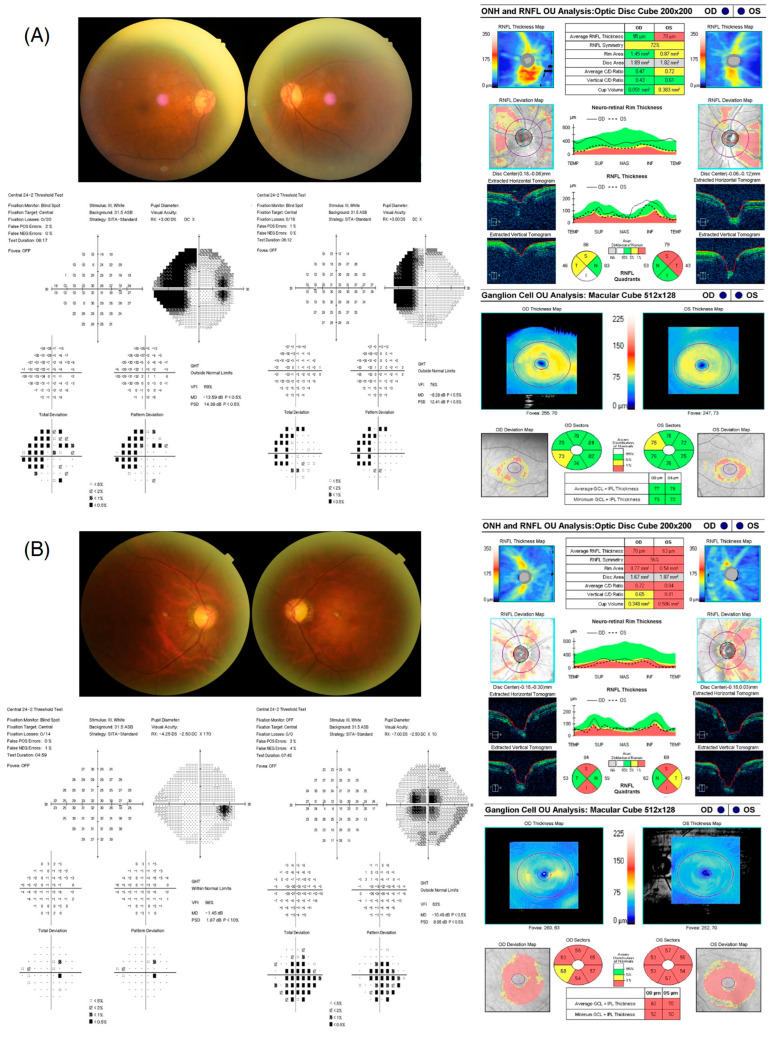
Representative cases of NTG-mimicking conditions and real NTG, both with atypical NTG characteristics. (**A**) The patient was a 72-year-old male with a VF defect that is suspected to respect vertical lines. The nasal sector value of the GCA thickness was 81.5 μm for the right eye, which was above the cut-off value. An old infarct was finally diagnosed in the right occipital lobe. (**B**) The patient was a 53-year-old male with a central VF defect. The nasal sector value of the GCA thickness was 53 μm for the left eye, which was below the cut-off value and eventually diagnosed as NTG without any specific findings in neuroimaging.

The ROC curves of individual parameters were plotted, and their AUCs and cut-off values were determined (Figure 1 and Table 4). Only GCIPL thicknesses at the nasal quadrant had reliable power for discriminating between eyes with neuro-ophthalmological disease and those with NTG (*p* = 0.003). For the GCIPL thickness at the nasal quadrant, the AUC was 0.659 and the cut-off value was 65.75. Therefore, in patients with features of atypical NTG, when the nasal sector’s GCIPL thickness is greater than 65.75 µm, neuroimaging can be strongly considered for discerning neuro-ophthalmological disease. In the current study, among the total of 36 cases diagnosed with neuro-ophthalmological disease, the nasal sector’s GCIPL thickness was thicker than the cut-off value of 65.75 in 21 cases (58.33%), while among the 104 cases of NTG patients, this was observed in 31 cases (29.81%). Figure 2 shows two representative cases with atypical NTG characteristics, and these cases show the usefulness of observing GCIPL thicknesses at the nasal quadrant in discriminating brain disease from NTG.

## 4. Discussion

In this study, among patients suspected of having a non-glaucomatous disease, 25.47% were diagnosed with conditions other than NTG, and the most frequent disease was meningioma. This aligns with findings from some studies in other countries where NTG is relatively uncommon, but there have also been other studies that reported higher or lower proportions [3,22,23]. This is thought to be attributed to variations in diagnostic criteria and factors such as race and age.

In the current study, among the reasons for undergoing neuroimaging, relatively fewer diagnoses of non-glaucomatous diseases were associated with unilateral NTG, the worsening of VA, and central scotoma. In typical NTG patients, it generally progresses in both eyes, with characteristic visual field defects such as nasal steps. However, in NTG patients with myopia, atypical visual impairment may occur by invading the central field, and progression may be more rapid in one eye depending on the degree of myopia [24,25]. On the other hand, factors such as rapid visual field progression, optic disc pallor, and scotoma restricted by a vertical line appear to be less influenced by the degree of myopia under the assumption of IOP regulation, resulting in a relatively higher diagnostic rate of non-glaucomatous diseases. This is noteworthy not only in South Korea, where myopia is prevalent in the majority, but also globally amid the increasing prevalence of myopia worldwide.

In this study, the highest rates of positive neuro-ophthalmological conditions were associated with factors such as rapid visual field progression and scotoma restricted by a vertical line, although the sample size was small. It is known that several systemic factors, such as blood circulation, may play a role in the pathogenesis of NTG, resulting in its comparatively slower progression rate of −0.36 dB per year. In contrast, POAG, which is primarily affected by high IOP, exhibits a faster progression rate of −1.31 dB per year [26]. Therefore, in cases of atypical NTG exhibiting rapid visual field loss greater than −1.5 dB per year, neuroimaging is essential to ensure that serious diseases are not overlooked.

In typical cases of NTG, visual field impairment is often initiated with nasal steps and progresses towards arcuate scotoma. This pattern of visual field loss in NTG is related to the direction of the optic nerve fibers. However, in the context of neuro-ophthalmological diseases that directly affect the visual pathway behind the eyeball, especially when compressive lesions press on the nerve, it can manifest as hemianopsia or quadrantanopsia that respects a vertical line regardless of the course of optic nerve fibers within the eye. Therefore, visual field damage that respects a vertical line can be considered a distinctive manifestation of neuro-ophthalmological diseases.

The most common reason for neuroimaging was unilateral involvement in NTG, yet the diagnosis rate for neuro-ophthalmological conditions was relatively low. Known for normal-range IOP and influenced by general conditions such as blood circulation, NTG typically progresses symmetrically in both eyes. However, the observed asymmetry in glaucoma progression among some patients suggests that, despite the general tendency for symmetrical progression, exceptions can occur relatively at the early stage.

The RNFL and nasal GCIPL thickness values were significantly different when comparing the glaucoma test results between NTG patients and those diagnosed with other neuro-ophthalmological conditions. It is known that visual pathway lesions are more likely to show GCLIPL abnormalities than compared to RNFL thicknesses [27]. This seems to be related to the trans-synaptic retrograde degeneration of retinal ganglion cells and the retinal nerve fiber layer following lesions in the visual pathway of the brain [28,29,30]. Degeneration at the posterior visual pathway, such as the presence of optic radiation lesions or occipital lobe injury, causes retinotopic changes to the anterior visual pathway from the brain to the eye [25,31]. However, the ratio of visual field damage to nerve damage did not show a statistically significant difference; this is likely due to the varying periods after the onset of the diseases.

Previous research findings also have indicated that diseases affecting the posterior visual pathway predominantly impact the nasal ganglion cells in the early stages, especially in compressive optic neuropathy [26,27]. In open-angle glaucoma, on the contrary, it is common for macular GCIPL defects to first appear in the temporal quadrant. This is due to the structure of the optic disc and the pathway of ganglion cells. This finding is already being utilized for glaucoma diagnosis in highly myopic patients where conventional glaucoma testing may be unreliable [17,32]. However, in this study, the nasal sector of GCIPL thicknesses was significantly thicker in patients with neuro-ophthalmological diseases compared to NTG patients. This finding is also consistent with a prior study targeting pituitary adenoma patients; in this study, the preperimetric pituitary adenoma group exhibited a significantly greater GCIPL thickness relative to the nasal sector compared to both the perimetric pituitary adenoma and NTG groups [12]. The reason for the thicker nasal part of the GCIPL in neuro-ophthalmological diseases compared to NTG may be attributed to the fact that in NTG, suspicion of neuro-ophthalmological disease arises when visual field damage has already progressed, resulting in the thinning of the nasal part. Moreover, NTG patients are less likely to undergo examinations initially due to the absence of symptoms, resulting in a relatively lower probability of hospital visits. In a real clinic setting, when encountering patients with atypical NTG characteristics, this finding can be helpful in determining whether neuroimaging should be strongly recommended.

Previous studies on NTG-mimicking diseases have not compared various glaucoma tests in real clinical settings while targeting a diverse range of conditions. Among the various atypical NTG characteristics that are already known, this study is significant in assisting clinicians in prioritizing tests and determining criteria for recommending neuroimaging when encountering patients with atypical NTG features in actual clinical practices.

Our study’s limitations include its small sample size. Therefore, there were limitations in determining the diagnostic rates of non-glaucomatous disease for each cause that led to undergoing neuroimaging. However, despite the small sample size, it was demonstrated to have sufficient statistical power, proving its reliability. Furthermore, the fact that the study was limited to a specific population at the hospital is also a limitation.

The sensitivity and specificity of GCIPL thickness at the nasal quadrant are indeed very low because the study included a variety of neuro-ophthalmological conditions rather than focusing on a specific one. Focusing on a specific condition, such as compressive optic neuropathy, can yield high sensitivity and specificity, but this has limitations in being helpful in real clinical settings. Therefore, this study included all patients diagnosed with any neuro-ophthalmological condition besides NTG. Furthermore, it is significant that meaningful results were obtained despite this heterogeneity.

In conclusion, when deciding whether to proceed with an imaging test such as MRI in an NTG patient with atypical NTG characteristics, it is advisable to consider the nasal sector cut-off value relative to GCIPL thicknesses. The objective of this study was not to overlook the existing criteria for performing imaging exams in atypical NTG cases. The fact that most imaging exams do not reveal diseases other than NTG suggests the need for additional reference metrics beyond the known characteristics of atypical NTG. This is particularly essential in the current context where the number of patients exhibiting atypical NTG patterns is increasing. Although nasal GCIPL thickness values have low specificity, it is meaningful that we have identified a potentially useful metric from existing glaucoma exam data that can be referenced before conducting imaging exams for patients with atypical NTG characteristics.

## Figures and Tables

**Table 1:** Comparison of demographic characteristics between patients with NTG and neuro-ophthalmological conditions.

Characteristics	Patients Diagnosed with Neuro-Ophthalmological Conditions (*N* = 27)	Patients Diagnosed with NTG (*N* = 79)	*p*-Value
Age	53.41 ± 16.70	51.22 ± 14.79	0.522
Gender (female/male)	16/11	45/34	
Laterality of unilateral glaucoma	6 right/13 left	21 right/33 left	
Components	Meningioma (*n* = 5) Pituitary macroadenoma *(n* = 2) Cavernous hemangioma (*n* = 1) Arachnoid cyst (*n* = 1) Aneurysm (*n* = 1) Rathke cleft cyst (*n* = 1) Pineal cyst (*n* = 1) Old infarct (*n* = 5) Intracranial hypertension (*n* = 1) Empty sella syndrome (*n* = 1) Optic neuritis (*n* = 4) Large Onodi air cell (*n* = 3) Orbital mass (*n* = 1)		

**Table 2:** Comparison of ocular characteristics between eyes of patients with NTG and neuro-ophthalmological conditions.

	Eyes with Atypical NTG Features		*p*-Value
	Eyes Diagnosed with Neuro-Ophthalmological Conditions (N = 36)	Eyes Diagnosed with NTG (N = 104)	
Age, year	54.69 ± 17.46 (17 to 80)	51.07 ± 15.03 (19 to 81)	0.234
IOP, mmHg	14.56 ± 3.23 (7 to 20)	14.47 ± 2.94 (8 to 21)	0.885
VA	0.69 ± 0.31 (0.01 to 1.0)	0.67 ± 0.32 (0.01 to 1.0)	0.746
AxL, mm	24.59 ± 1.60 (22.43 to 27.71)	24.76 ± 1.64 (21.51 to 28.52)	0.626
CCT, μm	523.47 ± 33.61 (438 to 588)	519.14 ± 81.97 (401 to 641)	0.765
C:D ratio	0.50 ± 0.21(0.1 to 0.8)	0.53 ± 0.21 (0.1 to 0.99)	0.486
**RNFL thickness average** **, μm**	**73.89 ±** **27.64 (39 to 215)**	**66.27 ±** **1** **2.85 (39 to 99)**	**0.029**
RNFL thickness temporal, μm	58.67 ± 24.85 (34 to 171)	52.71 ± 17.90 (26 to 128)	0.124
RNFL thickness nasal, μm	59.75 ± 12.29 (44 to 111)	58.39 ± 8.84 (29 to 87)	0.477
GCIPL thickness average, μm	65.56 ± 10.53 (49 to 91)	61.15 ± 12.09 (22 to 92)	0.282
GCIPL thickness temporal, μm	65.07 ± 11.18 (26 to 87)	61.29 ± 12.48 (25 to 84.5)	0.118
**GCIPL thickness nasal** **, μm**	**68.31 ±** **13.48 (46 to 98)**	**60.** **75 ± 1** **4.44 (11 to 100)**	**0.008**
MD, dB	−9.40 ± 7.90 (−29.38 to 1.22)	−8.89 ± 8.49 (−31.6 to 0.56)	0.756
VFI, %	74.97 ± 24.56 (4 to 100)	76.24 ± 26.59 (0 to100)	0.803
MD/RNFL thickness, dB/μm	−0.14 ± 0.14 (−0.57 to 0.02)	−0.15 ± 0.16 (−0.55 to 0.01)	0.834
MD/GCIPL thickness, dB/μm	−0.15 ± 0.14 (−0.52 to 0.02)	−0.16 ± 0.18 (−0.79 to 0.01)	0.704
VFI/RNFL thickness, %/μm	1.07 ± 0.38 (0.07 to 1.75)	1.17 ± 0.42 (0 to 2.1)	0.227
VFI/GCIPL thickness, %/μm	1.15 ± 0.41 (0.07 to 1.94)	1.25 ± 0.51 (0 to 2.86)	0.290
RNFL thickness temporal/nasal	0.99 ± 0.32 (0.55 to 1.57)	0.91 ± 0.30 (0.43 to 2.01)	0.190
GCIPL thickness temporal/nasal	0.97 ± 0.16 (0.45 to 1.33)	1.05 ± 0.30 (0.48 to 3.27)	0.149

Values are presented as mean ± SD. *p* < 0.05; IOP, intraocular pressure; VA, visual acuity; AxL, axial length; CCT, cornea central thickness; C:D ratio, cup–disc ratio; RNFL, retinal nerve fiber layer; GCIPL, ganglion cell–inner plexiform layer; MD, mean deviation; VFI, visual field index. Factors with statistical significance are shown in bold.

**Table 3:** Atypical NTG characteristics for which patients undergo neuroimaging.

Reason for Neuroimaging	Number of Patients (% of Total Patients)	Neuro-Ophthalmological Conditions (% of Patients Examined for This Reason)
Unilateral NTG	64 (59.81%)	18 (28.13%)
2. VF damage inconsistent with optic disc appearance	42 (39.25%)	14 (33.33%)
3. Fast visual field progression (1.5 dB per year or more)	5 (4.67%)	2 (40.00%)
4. Worsening of VA (≤0.5) (Worsening of VA of at least 2 lines in the Snellen chart connected neither with lens nor retinal pathology)	37 (34.58%)	11 (29.73%)
5. Optic disc pallor	37 (34.58%)	13 (35.14%)
6. Scotoma restricted by a vertical line	26 (24.30%)	10 (38.46%)
7. Central scotoma	19 (17.76%)	4 (21.05%)

NTG, normal-tension glaucoma; VA, visual acuity.

**Table 4:** Area under the receiver operating characteristic curves demonstrating the discriminating ability of the parameters for neuro-ophthalmological disease and NTG.

Parameters	Cut-Off Value	AUC (95% CI)	SEN	SPE	*p*-Value
Age	56.50	0.582	0.583	0.587	0.145
IOP	14.50	0.504	0.472	0.519	0.945
VA	0.62	0.511	0.722	0.375	0.839
CCT	529.50	0.457	0.441	0.447	0.450
AxL	24.76	0.465	0.448	0.550	0.569
C:D ratio	0.55	0.470	0.528	0.524	0.587
RNFL thickness average	65.50	0.601	0.556	0.538	0.073
RNFL thickness temporal	50.50	0.575	0.556	0.538	0.179
RNFL thickness nasal	57.50	0.492	0.444	0.471	0.883
GCIPL thickness average	63.50	0.607	0.559	0.598	0.063
GCIPL thickness temporal	64.75	0.594	0.588	0.592	0.102
**GCIPL thickness nasal**	**65.75**	**0.659**	**0.** **618**	**0.699**	**0.0** **06**
MD	−6.34	0.473	0.444	0.446	0.633
VFI	86.5	0.465	0.444	0.505	0.538
MD/RNFL thickness	−0.09	0.506	0.444	0.455	0.918
MD/GCIPL thickness	−0.09	0.510	0.471	0.505	0.857
VFI/RNFL thickness	1.19	0.416	0.444	0.485	0.137
VFI/GCIPL thickness	1.26	0.404	0.382	0.485	0.096
RNFL thickness temporal/nasal	0.90	0.573	0.583	0.596	0.194
GCIPL thickness temporal/nasal	1.00	0.436	0.382	0.485	0.261

AUC, area under the receiver operating characteristic curve; CI, confidence interval; SEN, sensitivity; SPE, specificity; IOP, intraocular pressure; VF, visual field; CCT, cornea central thickness; AxL, axial length; C:D ratio, cup–disc ratio; RNFL, retinal nerve fiber layer; GCIPL, ganglion cell–inner plexiform layer; MD, mean deviation; VFI, visual field index. Factors with statistical significance are shown in bold.

## Data Availability

The datasets generated and/or analyzed during the current study are available from the corresponding authors upon reasonable request.
